# The use of an educational video on forensic autopsy in curricular teaching for medical students—is it worth the effort? A field report from lecturers and a survey among students

**DOI:** 10.1007/s00414-023-03113-y

**Published:** 2023-11-09

**Authors:** Clara-Sophie Schwarz, Stefan Kramer, Tanja Germerott, Cleo Walz, Katrin Elsner

**Affiliations:** Institute of Forensic Medicine, University Hospital of Mainz, Am Pulverturm 3, 55131 Mainz, Germany

**Keywords:** Teaching in forensic medicine, Autopsy video, Emotions triggered by autopsy, Handling of corpses

## Abstract

**Purpose:**

The purpose of this work is to share our experience with an educational video on forensic autopsy. Using questionnaires, we attempted to answer the following questions: Does watching the video trigger emotions in students? Does the autopsy meet the expectations that they had before? Does the video help to prepare them for their subsequent autopsy participation?

**Methods:**

A total of 365 medical students who attended their classes during the COVID-19 pandemic measures were provided with the video on an online platform. Links leading to questionnaires were positioned before and after the video. One hundred seventy-six students returned to face-to-face teaching during their course in forensic medicine. Those among them who chose to participate in an autopsy at our institute were given the link to a third questionnaire after their autopsy participation. The data was analyzed using IBM SPSS 27.0 and Microsoft Excel.

**Results:**

One hundred seventy-two students completed a questionnaire before watching the educational video, 85 also completed one afterwards, and 28 completed the third questionnaire. The most intense feelings while watching the video were “curiosity” and “surprise”. Out of twelve students (14.1%) who had imagined the autopsy differently in advance, five perceived the autopsy shown in the video as rougher or more brutal than expected. All autopsy participants who had previously viewed the video felt adequately prepared.

**Conclusion:**

Teaching should include an introduction to the handling of the corpse and the general procedures in the dissecting room. Although a video cannot substitute for personal interaction, it is useful to prepare students for their autopsy participation.

## Introduction

Participation in forensic autopsies is a rare opportunity for medical students to gain autopsy experience outside of anatomy courses. Being familiar with the procedures of an autopsy helps clinicians decide in which situations an autopsy is to be recommended. Moreover, the autopsy itself is an opportunity where students can learn from the dead for the living. Last but not least, autopsy participation is important for those who are considering becoming forensic pathologists later on and want to get to know the professional field more thoroughly. Of course, it is also in the interest of forensic pathologists to attract interested young talents to their specialty.

At least in German-speaking countries, many students, unless they choose an elective internship in forensic medicine, attend very few, if any, autopsies during their studies apart from the anatomy course. It has been our experience that students are so impressed and distracted by experiencing the autopsy of a body without prior formaldehyde treatment that it becomes difficult to concentrate on teaching content. Therefore, students do not benefit in an ideal way from their autopsy participation. On the other hand, the autopsy physicians, who at the same time have the function of teachers, hardly have the capacity to respond to the students in the way that would be necessary in view of the sometimes obvious overstrain of the students. There is usually not enough time for long explanations or conversations during the autopsy itself as well as immediately before or after it. In our experience, a theoretical introduction by means of a lecture lacking the potential to show detailed video footage is only of limited use in preparing the students for their autopsy participation.

In a study by Tschernig et al. [1], 39% of all students surveyed said they wished to speak about death and related topics repeatedly during their anatomical dissection course. The majority preferred a “step-by-step contact” with the dead body. Twenty-three percent indicated that other students’ attitude or behavior towards the corpse was only partially correct; for 2% of the respondents, it was even incorrect. If students in the anatomy course want the handling of the dead human to be a topic, this is all the more to be expected in the context of teaching forensic medicine. Although the students are already further advanced in their studies at this point, the resemblance to the living body is more noticeable in the case of corpses that are not formalin-fixed and the autopsy process is also hardly comparable with an anatomical dissection. Thus, by no means is it likely that the uncertainties of handling corpses will have ceased to be a concern at this point.

Can mental overload also be a risk to students’ emotional well-being? Vicarious traumatization, according to McCann and Pearlman [2], is the development of psychological effects that can be painful and disruptive for individuals working with victims and can persist for months or years afterwards. The term was originally developed for psychologists serving victims or family members after traumatic events. Today, the term has broader applications, such as for soldiers after firefights or for police work. The findings regarding indirect traumatization can also be applied to the field of forensic medicine [3]. Most physicians in forensic medicine are likely to remember extraordinary cases well, even years later. This, for example, can be seen in books that recount the lives of forensic doctors and their most exciting cases (for example Working Stiff: Two Years, 262 Bodies, and the Making of a Medical Examiner [4]). But even in medical school, dealing with emotions while handling corpses is part of the socialization of future physicians [5].

Sergentanis et al. [6] asked students about their physical as well as psychological reactions witnessing an autopsy. They were able to identify five risk factors that led to “more adverse psychological reactions”:

- Female gender,
- Stereotypic beliefs about forensic pathologists,
- A more emotional frame of mind relative to forensic autopsy,
- More passive coping strategies,
- Greater fear of death.

Plaisant et al. [7] also described a significant difference related to gender with regard to the anatomic dissection course. Forty-eight percent of women and 18% of men had reported some form of anxiety prior to the anatomical dissection course. This anxiety decreased more in women over the duration of the course but also showed a higher baseline.

In a study by Papadodima et al. [8] students were asked if they could imagine a career in forensic medicine. Here, no significant difference was found between genders. A total of 26,5% of the students who could not imagine pursuing a career in forensic medicine stated, “Forensic doctors have a peculiar character”, whereas only 3.8% of the others said the same. In a survey by Hanzlick et al. [9], forensic pathologists were asked what motivated their choice of specialty. The second most important motivator cited was the influence of a mentor or professor. Thus, the impression forensic pathologists make on students appears to play an important role in the choice of specialty. According to Wright et al. [10], the influence of role models is the main reason for choosing a specialty. This fact should be another motivation to teach students about forensic autopsy in an appropriate way. But how can this be ensured in view of the always limited time and personnel resources?

The purpose of this work is to present our experience with a self-directed educational video on forensic autopsy in medical teaching. Originally, the video was made to give students the opportunity to experience a forensic autopsy during the COVID-19 pandemic measures despite the cancellation of face-to-face teaching. After the reintroduction of face-to-face teaching, we used the same video to prepare medical students to participate in a forensic autopsy.

Using questionnaires, we attempted to answer the following questions in relation to the autopsy video: Does watching the autopsy video trigger emotions in students? If so, which ones? Does the autopsy meet the expectations that the students previously had of a forensic autopsy?

In the second step, we surveyed students who were allowed to return to face-to-face classes after the strict pandemic measures ended and whom we prepared with the autopsy video. The essential question here was whether the autopsy video as an intermediate step between lecture and autopsy participation helps to better prepare the students for their autopsy participation.

## Material and methods

The sample consisted of the participants of the lecture course in forensic medicine in the summer semester of 2021 and the winter semester of 2021/22 of the Johannes Gutenberg University in Mainz. This is a compulsory course for fourth year students. The students were provided with the autopsy video on an online learning platform. It could be watched optionally. Links leading to online questionnaires were positioned before as well as after the video. The questionnaires were located on the website of the provider “LimeSurvey”. The data was sent via an encrypted SSL connection. No personal data that would allow conclusions to be drawn about the participants of the questionnaire were collected. The IP addresses were not stored.

The autopsy video was only released during the course when basic theoretical knowledge had already been taught. The video is 1 h, 1 min, and 20 s long and shows a complete autopsy. The deceased had volunteered to be a body donor for medical teaching during her lifetime. Thus, the autopsy depicted in the video is not an actual forensic autopsy, but a teaching autopsy intended to show how a forensic autopsy proceeds. The autopsy is preceded by a short introduction about the content that follows as well as a brief explanation of the general conditions of a forensic autopsy. This is followed by a note that blood, bodily fluids, unpleasant sounds, etc. are depicted in the video and that there is no obligation to watch the autopsy video. Of course, it is also pointed out that the reproduction or distribution of the video is prohibited. At minute 3:32, the body of the deceased can be seen for the first time. The face as well as the genitals are covered. The autopsy is performed according to the usual standard. There was no script. One specialist was assigned as a commentator and moderator.

**Fig. 1:**
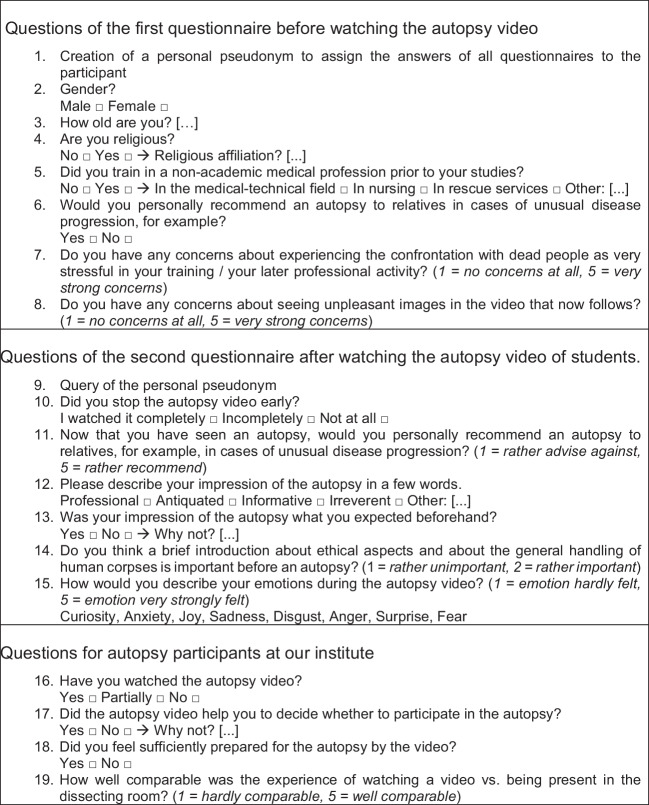
List of all questionnaire items evaluated in this paper

The questionnaires used are self-developed, non-standardized questionnaires. They also included questions that were used for internal teaching evaluation, for example, and have not been addressed here. The questions that were enclosed in this study are listed in Fig. 1.

From the first questionnaire, eight items were evaluated here. The first part of the first questionnaire asked demographic questions (age, gender, religiosity, previous experience), and the second part asked questions about expectations regarding autopsies. Response formats consisted of single-choice, multiple-choice, Likert scales, and free-text response options.

From the second questionnaire, seven items were evaluated here. This time the questionnaire did not contain demographic questions, but only questions about impressions while watching the autopsy video.

Some of the group (winter semester 2021/2022) were allowed to return to face-to-face teaching after the strict pandemic measures were terminated. Participation in a forensic autopsy was made possible for volunteers. These students were given the link to a third questionnaire after their participation. Only four items from this questionnaire were considered here. These questions addressed the students’ impressions during autopsy participation and their preparation using the autopsy video.

The group in the summer semester of 2021 (189 students) did not have the opportunity to participate in an autopsy at our institute. The group in the winter semester of 2021/22 (176 students) could both access the video and participate in an autopsy. Accordingly, the group that could potentially have completed the third questionnaire was significantly smaller than the group that was asked to complete the first two questionnaires.

Only fully completed surveys were evaluated. Data were evaluated and analyzed using IBM SPSS 27.0. The Mann-Whitney-U test was used to calculate statistical significance. The significance level was set at 5%. Significance testing was not performed for the third questionnaire because of the small sample size.

## Results

### Evaluation of the questionnaire to be completed *before* watching the autopsy video

#### Demographic features (Questions 2–5)

From the group invited to the survey (365 students), 169 participants accessed the first questionnaire (before watching the video), of which 127 completed the questionnaire in full (response rate 37,79%). Out of these, 79 participants are women (62.2%) and 47 participants are men (37%). One person did not specify a binary gender identity. The average age was 26.32 years, with 25% younger than or exactly 24 years old and 25% older than or exactly 29 years old at the time of the survey.

Sixty-four participants (50.4%) reported being non-religious, 56 participants (44.1%) were religious, of which 25 participants reported being Catholic, 23 participants were Protestant, and two participants reported being Christian. One person was Muslim and five participants were religious with no indication of their faith. Seven participants abstained when asked about their religious affiliation.

Ninety-two participants (72.4%) had already worked in a non-academic occupation prior to their studies. Out of these, 31 students (33.7%) had worked in rescue services, 28 (30.4%) in nursing, 14 (15.2%) in the technical field, and 19 participants (20.7%) indicated another occupational field. Multiple answers were possible.

#### Attitudes and expectations (Questions 6–8)

Before watching the autopsy video, 122 people (96.1%) indicated that they would recommend an autopsy to relatives in cases of unusual disease progression.

The question “Do you have any concerns about experiencing the confrontation with dead people as very stressful in your training / your later professional activity?” was rated by the students with an average of 2.07 points out of a maximum of 5 possible points. Here, a score of 1 is considered no concern at all and a score of 5 is considered a very strong concern. There is a statistically significant difference in mean scores for this question related to religiousness. Individuals who reported being religious, with a mean of 2.20, were said to have more concerns about experiencing the confrontation with dead people as stressful than individuals who did not report being religious (mean 1.89). The *P* value obtained by the Mann-Whitney-U test is 0.049. Thus, the result is below the pre-determined alpha significance level of 5%. Differences are also evident in relation to gender. Females reported a mean value of 2.22, while male participants reported 1.83. The *P* value is 0.012. Thus, the difference obtained is also statistically significant. Non-academic training before studying medicine also seems to play a modulating role. The arithmetic mean is 2.4 without non-academic training and 1.95 with non-academic training. The *P* value is 0.018. This difference therefore is statistically significant, too (Fig. 1).

In response to the question “Do you have any concerns about seeing unpleasant images in the video that now follows?”, the mean score is 2.17 out of a maximum of 5 possible points. A score of 1 is considered to mean “no concerns at all” and a score of 5 is considered to mean “very strong concerns”. Similar to the previous question, the answers show a relevant difference with regard to religiosity. For the religious, the mean value is 2.48, and for the non-religious 1.92, with a *P* value of 0.009. Likewise, there is a difference in the mean values of women and men. For women, the mean value is 2.37, and for men 1.83. With a P-value of 0.012, the difference is statistically significant. The influence of completed non-academic training before studying medicine is reflected as follows: with non-academic training, there is a mean value of 2.13, and without non-academic training 2.26. The *P* value is 0.822, so the difference is not statistically significant in contrast to the previous question.

### Evaluation of the questionnaire to be completed *after* watching the autopsy video (Questions 10–15)

Out of a total of 89 students accessing the questionnaire after watching the autopsy video, 85 completed the questionnaire in full (response rate 23,29%). Out of these, 81 people (95.3%) watched the autopsy video in its entirety and four people (4.7%) watched only parts of it.

Also, after viewing the autopsy video, students again were asked whether they would recommend an autopsy to relatives in cases of unusual disease progression. Eighty-two people (96.4%) stated to tend to recommend an autopsy to their relatives (score greater than 3). The arithmetic mean is 4.34 on a five-point scale, where 1 means “rather advise against” and 5 means “rather recommend”.

The impression of the autopsy as a medical examination method was as follows: 76 participants (89.4%) perceived the examination method as professional, 66 persons (77.6%) as informative, five persons (5.9%) as irreverent, and one person (1.2%) as antiquated.

We asked the students if their impression of the autopsy was as they had expected it beforehand. This question was answered in the affirmative by 66 students (77.6%). Seven students (8.2%) abstained from answering. Among the twelve students (14.1%) who answered the question in the negative, it is striking that five (5.9%) perceived the autopsy to be rougher or more brutal than they expected.

In response to the question “Do you think a brief introduction about ethical aspects and about the general handling of human corpses is important before an autopsy?” the arithmetic mean on the five-point scale is 4.48, and 74 people (87.1%) think an introduction is important (score greater than 3).

**Fig. 2:**
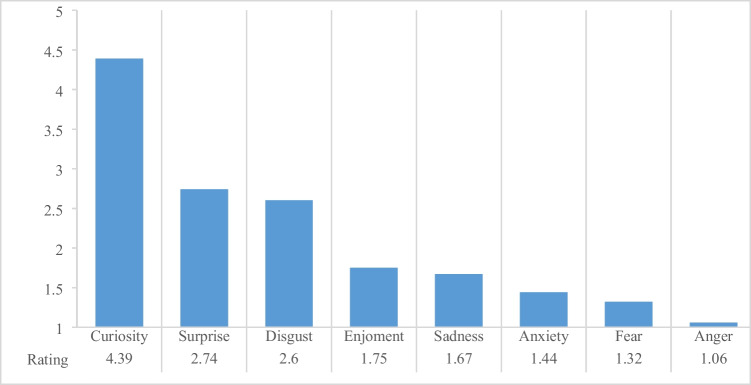
Mean values for the emotional experience while watching the autopsy video. The students were asked to indicate the intensity of different emotions when watching the autopsy video on a five-point scale. A score of 5 means “feeling the emotion very strongly”, and a score of 1 means “hardly feeling the emotion”

The participants were also asked questions about their emotional experiences watching the autopsy video. For this purpose, the students had to indicate the intensity of the emotions felt (curiosity, anxiety, enjoyment, sadness, disgust, anger, surprise, fear) on a five-point scale. A score of 5 means “very strong sensing”, and 1 means “hardly any sensing of the emotion”. The mean values of this five-point scale can be seen in Fig. 2. The most intense feelings were curiosity and surprise. After all, while watching the video, nine persons felt “disgust” strongly or very strongly, six persons felt “sadness” strongly, four persons felt “anxiety” strongly or very strongly, one person felt “anger” very strongly and one person felt “fear” very strongly.

Participants who expressed that they were more concerned about either seeing unpleasant images in the autopsy video (20%) or having contact with corpses later in their careers (8.2%) now showed higher scores for the emotions “anxiety” (2.17 and 1.71, respectively), “disgust” (3.50 and 3.14, respectively), and “fear” (1.58 and 1.43, respectively). The emotions “curiosity”, with mean scores of 4.00 and 4.29, and “surprise”, with 2.33 and 2.14, respectively, were lower than in the overall group.

### Evaluation of the questionnaire to be completed after autopsy participation in our institute (Questions 16–19)

The questionnaire after participation in an on-site autopsy at the Institute of Forensic Medicine was filled out completely by only 28 students. A response rate cannot be given here since autopsy attendance is optional and only those students who actually attended an autopsy were given the questionnaire. Anyway, it was observed that not all participants took time to complete the questionnaire after their autopsy participation. Of those who completed the questionnaire, 25 individuals had viewed the autopsy video in full (23) or in part (2) in advance of their autopsy participation.

Twenty-two participants stated that the autopsy video helped them decide whether to participate in an autopsy at our institute. The three people who answered this question in the negative explained that they had already decided to participate beforehand.

**Fig. 3:**
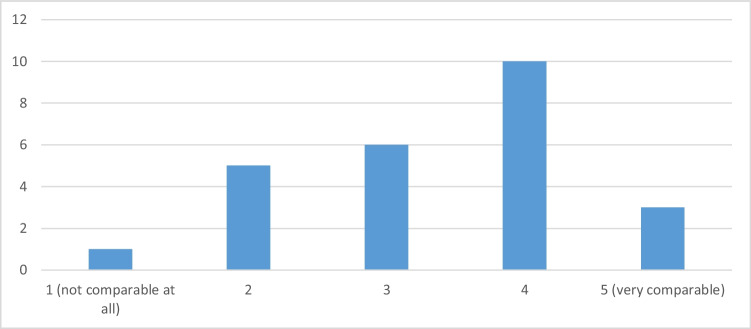
Responses to the question “How comparable was the experience of watching a video vs. being present in the dissecting room?”. Just over half of those who watched the video think that watching the video was well comparable with the autopsy participation (score greater than 3)

All 25 autopsy participants who had previously viewed the video in whole or in part felt that the video adequately prepared them for the autopsy. In response to the question “How well comparable was the experience of watching a video vs. being present in the dissecting room?” the arithmetic mean on the five-point scale is 3.36, and 13 people (just over half of those who watched the video) think that watching the video was well comparable with the autopsy participation (score greater than 3), as shown in Fig. 3.

## Discussion

Before watching the autopsy video, 96.1% of the participating students indicated that they would recommend an autopsy to their relatives in cases of unusual disease progression. Afterwards, even 96.4% would tend to recommend an autopsy to their relatives. This indicates that a lege artis performed autopsy per se is not perceived as problematic with regard to the dignity of the corpse, which is consistent with other studies [11]. It also suggests that students approach autopsy as an examination method with an open mind and, when the procedures are explained to them, predominantly perceive autopsy as a valuable tool in medicine. Accordingly, 89.4% stated that they experienced autopsy as a professional examination method. However, the fact that 5.9% perceived the autopsy as rougher or more brutal than previously expected indicates that an adequate theoretical introduction and accompanying explanations are necessary. Accordingly, a large majority is of the opinion that an introduction to ethical aspects and to the handling of the corpse in general is important. This result is basically in agreement with the results of Tschernig et al. [1] for the anatomical dissection course, that was mentioned in the introduction of this article, although in our study there was an even more explicit emphasis on addressing the handling of the corpse when teaching students.

Concerns about being less able to withstand confrontation with dead people in the personal medical career and while watching the video were expressed to a significantly greater extent by women, similar to the study by Plaisant et al. [7], and religious individuals. Non-academic training resulted in lower scores on concerns about confrontations with dead people in medical careers and while watching the video, although the latter was not statistically significant. Participants who expressed that they were more concerned about having contact with corpses or about watching the video showed higher scores for the emotions “anxiety”, “disgust”, and “fear” while watching the video. This can be taken as a further indication of how important an appropriate introduction is not only for the presentation of the discipline to the outside world but also for the emotional well-being of the students.

It is well known that educational videos can be useful tools in clinical and forensic teaching [12, 13]. But can an educational video help to prepare students for autopsy participation? In our study, all autopsy participants felt well prepared by the video. A certain comparability of the video with an autopsy participation was confirmed. This can be an advantage for learners and teachers since the limited time in the autopsy room can be used more effectively and the students feel more comfortable because they know concretely what to expect. A disadvantage could be that students feel distressed by watching the autopsy video while there is not a specialist available to address their feelings as would be the case in face-to-face teaching. From a survey among students receiving online education in forensic medicine, we know that the preferred approach to potentially distressing content varies. Some prefer to explore such content on their own, allowing them to process their feelings in private, while others prefer to approach the material with their peers [14]. However, from our point of view, the danger of distressing students by letting them watch an educational video is lower than distressing them in the autopsy room with only theoretical preparation through lectures, because everything was aligned for teaching purposes in the video, while in the daily routine work has to be done under time pressure which results in scant time for explanations along the way. In addition, when watching the video, it is possible to press the stop button, whereas in the autopsy room, it may be difficult to escape the situation due to e. g. embarrassment in front of fellow students. Moreover, we received consistently positive feedback from the students for the video, so we assume that the video does more good than harm.

Regarding the questions about emotions triggered by a forensic autopsy in medical students, there is a methodological advantage to using the autopsy video. The advantage is that in the video all students saw one and the same autopsy, rather than one group watching the autopsy of a deceased child and the other group watching the autopsy of an elderly person, for example. Nevertheless, the feelings experienced while watching an educational video do not fully translate to the feelings experienced while participating in an autopsy, of course. This is also the reason why we think the video could be a good intermediate step.

Methodologically problematic is the investigation of the question to what extent the autopsy video is suitable as a means of preparation for autopsy participation. The sample of autopsy participants is much smaller since the autopsy participation was voluntary and a greater effort than watching a video, and the students were apparently less motivated to fill out a questionnaire after their autopsy participation. A comparison to the impressions students had of their autopsy participation without being prepared by watching the video is not possible, as no survey was conducted in the time before the availability of the video. Thus, the validity of this part of the study is limited. Nevertheless, it is at least possible to see that, in retrospect, the students who came forward to participate in the autopsy after watching the video felt well prepared by the video.

Furthermore, it can be assumed that some of the students who watched the video decided not to participate in an autopsy based on the video. Thus, one advantage of the video provided in advance may also be that only students who are confident to participate while knowing the procedures and who are particularly interested show up for the autopsy so that teaching in the autopsy room can be arranged in a beneficial way. Because obligatory autopsy attendance is not intended at our institution, students who decided not to attend could not be recorded separately in our study design. Thus, they also could not be asked about the reasons why they did not come to the autopsy room. In a future study, to further evaluate the video, we could try to reach these students in another teaching module that is obligatory and have them fill out a questionnaire.

Overall, we were able to show that teaching in forensic medicine should include an introduction to handling the human corpse and general procedures in the dissecting room. This is important in order to present our discipline appropriately and to ensure the emotional well-being of students. Even if an educational video does not replace the direct approach to the students, in our experience it can be a useful tool to prepare students for their experiences in the autopsy room.

## Declarations

This study does not include medical data collected from patients or corpses. The Research Ethics Committee of the Rhineland-Palatinate Medical Association has confirmed that no ethical approval is required.

This article does not contain any studies with animals performed by any of the authors.
